# Household level of air pollution and its impact on the occurrence of Acute Respiratory Illness among children under five: secondary analysis of Demographic and Health Survey in West Africa

**DOI:** 10.1186/s12889-022-14611-w

**Published:** 2022-12-12

**Authors:** Mouhamadou Lamine Daffe, Salimata Thiam, Fatoumata Bah, Awa Ndong, Mathilde Cabral, Cheikh Diop, Aminata Toure, Absa Lam, Mamadou Fall

**Affiliations:** 1 Toxicology and Hydrology Laboratory, Faculty of Medicine, Pharmacy and Dentistry, Cheikh Anta Diop University, Fann, Avenue Cheikh Anta Diop, PoB:5005, Dakar, Senegal; 2 Department of Physical and Chemical Sciences, Training and Research Unit of Health Sciences Iba Der Thiam University, Grand Standing, PoB: A967, Thiès, Senegal

**Keywords:** Indoor air pollution, Combustion processes, African Economic and Monetary Union area, Acute Respiratory Illness, Children under-five, Demographic and Health Survey

## Abstract

**Background:**

One out of ten deaths of children under five are attributable to indoor air pollution. And Acute Respiratory Illness (ARI) is among the direct causes.

**Objective:**

This study showed the possibilities of characterizing indoor air pollution in West African Economic and Monetary Union (WAEMU) area and it also made it possible to estimate its impact on the occurrence of ARI in children under five.

**Methods:**

It has been a secondary analysis based on Demographic and Health Surveys (DHSs) from WAEMU countries’ data.. “*Household level of air pollution*” is the created composite variable, from questions on the degradation factors of indoor air quality (domestic combustion processes) which served to characterize indoor air pollution and to measure its impact by a logistic regression.

**Results:**

Burkina Faso stands out with a greater number of households with a high level of pollution (63.7%) followed by Benin (43.7%) then Togo (43.0%). The main exposure factor "*Household level of air pollution*" was associated with ARI symptoms (Togo: prevalence = 51.3%; *chi-squared* test’s *p-value* < 0.001). Exposure to high level of pollution constitutes a risk (AOR [95 CI]), even though it is not significant ( Ivory Coast: 1.29 [0.72–2.30], Senegal: 1.39 [0.94–2.05] and Togo: 1.15 [0.67–1.95]) and this could be explained by the high infectious etiology of the ARI.

## Introduction

Polluted air corresponds to a heterogeneous mixture of chemical compounds in gaseous (NOx, CO, O3, etc.) or particulate form, which particles are adsorbed by organic and inorganic substances and classified according to their aerodynamic diameter (PM_10_, PM_2.5_, etc.) [1, 2]. This pollution come from various sources, natural as well as anthropogenic. In Africa, anthropogenic sources are concentrated near or even inside dwellings. In fact, a large part of households use rudimentary means (solid fuels, open fireplaces in living rooms) in cooking activities, thus highly polluting indoor air [3–7]. Several other households still depend on polluting fuels for their lighting needs, and are also exposed to other factors that affect indoor air quality, such as smoking and waste incineration [5, 8]. In addition, there are also the frequent use of incense, demographic pressure, and the influence of urban traffic [5]. This pollution is the underlying cause of 4 million deaths per year, the tenth of which occur in children under five, and low and middle incomes countries (LMICs), especially those in Africa are the most impacted [9]. Acute Respiratory Illness (ARI), are among conditions the most associated with this high infant mortality [10]. In addition, the prevalence of symptoms of ARI is 4 times higher in Africa compared to Europe, and the risk is multiplied by 3 in children exposed to solid fuel smoke [4, 11, 12]. However, in Africa, and more particularly in the West African Economic and Monetary Union (WAEMU) area, few studies exist on indoor air quality [4, 5, 13–15]. The majority of these studies reveal levels of particulate pollution (PM_10_ and PM_2.5_) well above the WHO guideline values, which is the case of Senegal with PM_10_ concentrations estimated at 200 μg/m^3^ and in Burkina Faso with PM_2.5_ concentrations estimated at 26.5 mg/m^3^. Therefore, it appears justified to find approaches aimed at filling this gap, hence this present work, which has objectives of characterizing indoor air pollution in the WAEMU area and estimating its impact on the occurrence of ARI symptoms.

## Methods

### Setting and study design

This is a retrospective cross-sectional study, in which we carried out a secondary analysis on data from DHS conducted in WAEMU member states. WAEMU is composed of eight Sahelian countries, linked by a common currency and cultural traditions: these are Benin, Burkina Faso, Mali, Niger, Ivory Coast, Guinea-Bissau, Senegal and Togo [16, 17]. It covers an area of 3.5 million km^2^ and has more than 120 million inhabitants, 34.8% of whom live in urban areas with disparities between countries [18, 19]. Indeed, urban population is larger in Ivory Coast (53.8%), Senegal (46.5%) and Benin (44.6%) and lower in Niger (14.9%). In addition, Ivory Coast represents 20.6% of the total population of the area, followed by Niger with 17.3% [19]. WAEMU area faces challenges related to poverty, access to basic social services, high fertility and is characterized by a high infant mortality [19]. In sum, the study included 59,765 children (Benin: 12,432; Burkina Faso: 13,583; Ivory Coast: 6941; Mali: 9222; Senegal: 11,182; and Togo: 6405), and 65,705 households (Benin: 14,156; Burkina Faso: 14,424; Côte d'Ivoire: 9686; Mali: 9510; Senegal: 8380; and Togo: 9549).

### DHS data sampling and collection

DHS are designed to be nationally representative and aimed to provide information on the characteristics of the population (family planning, maternal and child health, child survival status, HIV/AIDS, Sexually Transmitted Infections (STIs), reproductive health, nutritional status, etc.). Data were collected according to a complex multi-stage stratified cluster sampling design. At first, Enumeration Areas (EAs) were identified and then drawn from a list established during the last General Population and Housing Census (RGPH), then in each selected EA, a sample of households was drawn from an updated list. Survey participants included women aged 15 to 49, men aged 15 to 59, and children under five. As regards to the latter, their mothers were invited to provide information on their demographic characteristics as well as their health status. Four questionnaires were used for data collection: household questionnaire, female questionnaire, male questionnaire and biomarker questionnaire. Household questionnaire served as a tool for collecting information on household characteristics (main source of drinking water, type of toilet, hand washing equipment, source of lighting, fuels and cooking place, passive smoking, etc.). It also allowed to identify household members eligible for individual interviews and/or biological tests and measurements. A Biomarker questionnaire allowed informing the anthropometric measurements as well as results of tests carried out on blood samples [20–26]. Results presented in this paper are based on characteristics of households and children under five included in the sixth DHS (Burkina Faso, Ivory Coast) and seventh DHS (Benin, Senegal, Togo, Mali). Databases were obtained following a request and a justification of study from managers of the DHS program. Guinea Bissau is not concerned by the DHS program and is therefore excluded, as is Niger due to the unavailability of some variables of interest in the used database.

### Operational description of variables

“*Household level of air pollution*” is the created composite variable, from questions on the degradation factors of indoor air quality (domestic combustion processes) which served to characterize indoor air pollution and to measure its impact by a logistic regression. These questions were: “*Does your household have electricity?”; “What type of fuel does your household mainly use for cooking?”; “Is the cooking usually done in the house in a separate building or outdoors?”; “How often does anyone smoke inside your house, would you say daily, weekly, monthly, less often than once a month, or never?”; “Do you currently smoke cigarettes every day, some days, or not at all?*”. The possible answers to some of these questions were first grouped before being assigned a score. As regards to the type of cooking fuel, grouping is based on the work of Mishra et al. [27]. Three categories corresponding to high pollution fuels (*wood, straw/shrubs/grass, agricultural crop or animal dung*), medium pollution fuels (*Kerosene, coal/lignite or charcoal*), and low pollution fuels (*electricity, Liquefied Petroleum Gas, natural gas, biogas*) are indeed defined on the basis of the answers to this question. The scores assigned to these categories were 3, 2 and 1 respectively. The question on the smoking status of household members was also categorized into three modalities (*never, sometimes and daily*) with scores of 0, 1 and 2 respectively. “*sometimes*” was introduced as a new modality and includes the following responses: *weekly, monthly, and less often than once a month*. Concerning the mothers’ smoking status, the variable was binarized (*yes/no*) by regrouping under the “*yes*” modality, the following answers: *every day* or *some days*. Thus, the score assigned to this variable was 1 for “*yes*” and 0 for “*no*”. The same is applied to the availability of electricity, which was collected in a binary form. Also, the place of cooking was not recoded, and the answers were *outdoors*, *in a separate building* or *in the house*, corresponding respectively to the following scores: 1, 2 and 3. The maximum summation of the scores is 10. Subsequently, three levels of scores were defined for “*Household level of air pollution*”: *low level* corresponding to households with a score less than 4; *medium level* for those with a score between 4 and 6; and *high level* when the score is greater than 6. The second variable of interest is defined by symptoms of ARI and is used to characterize respiratory health of children. The definition proposed for this indicator has evolves over time and this work retained the DHS Statistics Guide’s latest definition. Symptoms of ARI in the child is defined as “*short, rapid breathing which was chest-related and/or difficult breathing which was chest-related*” during two weeks preceding the survey [28]. These symptoms were self-reported by children’s mothers. Moreover, DHS Statistics Guide also classified types of drinking water and sanitation facilities into one of the following: *improved* and *unimproved* [28]. This classification is based on guidelines of WHO/UNICEF’s Joint Monitoring Program for water supply and sanitation [29].

### Statistical analysis

We did a a frequency measurement to describe households and children included in the study. The two main variables used for this purpose are: “*Household level of air pollution*” and symptoms of ARI. In addition to variables used to construct these indicators, other variables were included in this phase of the analysis. For households, the latter variables are relating to the access of water, hygiene and sanitation, as for children, they are: age, sex, birth weight and the mothers’ age and level of education. The second phase of analysis was carried out by measures of association using the *chi-squared* test and logistic regression. At this stage, symptoms of ARI are defined as dependent variable and “*Household level of air pollution*” as the main exposure factors. Other variables used in households and children’s description steps were also taken into account. A multivariate logistic regression model was fitted by including all variables significantly associated with the occurrence of ARI in any of the WAEMU member countries according to *chi-squared* test’s *p-value*. Adjusted Odds Ratios (AOR) were estimated from regression models as well as 95% confidence intervals (95% CI). All statistical analyses were carried out using the *R* software. The “*survey*” package is used to weigh all the observations in order to compensate for the oversampling of certain categories of respondents and to take into account the complexity of the sampling plan.

## Results

### Household characteristics

The study reveals a predominance of rural households except for Senegal which has 48.3%. In addition, the lowest levels of urbanization are found in Mali (22.3%) and Burkina Faso (24.9%). Poverty (*poorest* + *poor*)/wealth (*richest* + *richer*) ratio is less than 1 in WAEMU area, except for Ivory Coast, for which the number of households classified as rich (20.6% + 18.1% = 38.7%) is slightly lower than the number of poor households (21.3% + 18.3% = 39.6%), reflecting a higher number of households with a good standard of living. The overwhelming majority of households except those in Senegal use highly polluting fuels for cooking (Benin: 65.9%; Burkina Faso: 87.7%; Ivory Coast: 60.2%; Mali: 77.9%; Togo: 49.4%). In fact, in these countries, wood (Benin: 61.9%; Burkina Faso: 87.5%; Ivory Coast: 60.1%; Mali: 77.6%; Togo: 1.1%) and charcoal (Benin: 26.7%; Burkina Faso: 4.3%; Ivory Coast: 17.6%; Mali: 19.3%; Togo: 48.2%) are the main cooking fuels. In addition, a more accentuated use of lignite is observed in Togo (42.1%) and Senegal (43.1%), the latter being also characterized by a higher number of households using liquefied petroleum gas (27.0%). In most of these countries, cooking is done mainly outdoor (Benin: 50.6%; Burkina Faso: 67.9%; Ivory Coast: 50.6%; Togo: 66.6%). However, in Mali, it mainly takes place at home but in a separate room (68.8%) while in Senegal, it mostly takes place in the housing (71.3%). In terms of access to electricity, highest rates are noted in Senegal (63.3%) and Ivory Coast (55.9%) while in other countries, more than half of households do not have access (Benin: 64.4%; Burkina Faso: 86.8%; Mali: 51.4%; Togo: 54.2%). Although the majority of households are not exposed to passive smoking, prevalence of daily exposure varies between 8.5% (Benin) and 21.8% (Ivory Coast). In most of the countries, drinking water comes mainly from improved sources (Benin: 71.7%; Burkina Faso: 77.0%; Ivory Coast: 78.4%; Mali: 69.3%; Senegal: 85.1%; Togo: 67.8%). As regards to access to sanitation, disparities are noted between countries. In fact, in Senegal and Ivory Coast, more than half of households have access to improved toilets (Senegal: 72.7%; Ivory Coast: 55.4%) while in Benin and Burkina Faso, it is the practice of open defecation that predominates (Benin: 53.9%; Burkina Faso: 62.3%). Concerning access to hygiene, except Senegal, more than half of households in WAEMU area have a place dedicated to handwashing (Benin: 55.1%; Burkina Faso: 80.9%; Ivory Coast: 87.0%; Mali: 71.2%; Togo: 83.7%) (cf. Table 1).

**Table 1:** WAEMU countries Household characteristics (in %) including domestic energy, access to water, hygiene and sanitation

Household characteristics	Benin	Burkina Faso	Ivory Coast	Mali	Senegal	Togo
	*N* = 14,156	*N* = 14,424	*N* = 9686	*N* = 9510	*N* = 8380	*N* = 9549
Type of place of residence						
n	14,156	14,424	9686	9510	8380	9549
urban	43.1	24.9	45.4	22.3	51.7	44
rural	56.9	75.1	54.6	77.7	48.3	56
Wealth Index Combined						
n	14,156	14,424	9686	9510	8380	9549
poorest	17.7	19.4	21.3	19.2	18.8	12.6
poorer	18.9	20.2	18.3	20.0	17.6	17.3
middle	19.8	19.3	21.7	20.5	19.5	22.9
richer	20.8	19.2	20.6	20.9	23.0	24.5
richest	22.8	21.9	18.1	19.4	21.1	22.7
Type of cooking fuel						
n	14,156	14,424	9678	9510	8380	9548
electricity	0.3	0.0	0.0	0.5	1.3	0.0
liquefied petroleum gas	3.3	4.6	14.8	0.3	27.0	6.5
natural gas	1.2	0.4	0.2	0.3	0.0	0.1
biogas	0.2	0.4	0.0	0.0	0.0	0.2
Kerosene	0.2	0.0	0.0	0.0	0.0	0.0
coal/lignite	1.0	0.1	0.2	0.4	43.1	42.1
Charcoal	26.7	4.3	17.6	19.3	18.9	48.2
Wood	61.9	87.5	60.1	77.6	0.0	1.1
straw/shrubs/grass	3.9	0.1	0.2	0.0	0.0	0.1
agricultural crop	0.1	0.0	0.0	0.0	0.4	0.0
animal dung	0.0	0.0	0.0	0.3	1.5	0.0
no food cooked in house	1.0	2.6	6.9	1.4	7.6	1.6
other	0.2	0.0	0.1	0.0	0.0	0.0
low pollution fuels^a^	5.0	5.3	15.0	1.1	28.4	6.9
medium pollution fuels^b^	27.9	4.4	17.8	19.6	62.1	42.3
high pollution fuels^c^	65.9	87.7	60.2	77.9	1.9	49.4
Where food cooked in the house						
n	14,007	14,043	8992	9376	7881	9404
in the house	34.6	8.8	11.3	3.5	71.3	9.6
in a separate building	14.0	23.2	37.8	68.8	21.1	23.7
outdoors	50.6	67.9	50.6	27.4	7.2	66.6
other	0.8	0.1	0.3	0.3	0.4	0.0
Household has electricity						
n	14,156	14,424	9678	9510	8380	9543
yes	35.6	13.1	55.9	48.6	63.3	45.8
no	64.4	86.8	44.1	51.4	36.7	54.2
don’t know	0.0	0.1	0.0	0.0	0.0	0.0
Main source of drinking water						
n	14,156	14,424	9679	9510	8380	9542
unimproved	28.3	23.0	21.6	30.7	14.9	32.2
improved	71.7	77.0	78.4	69.3	85.1	67.8
Location of the source of water						
n	10,569	13,348	6789	7744	3926	8951
in own dwelling/yard/plot	17.2	3.8	18.6	17.5	14.6	10.5
elsewhere	82.8	96.2	81.4	82.5	85.4	89.5
Type of toilet facility						
n	14,156	14,424	9664	9510	8380	9544
unimproved	11.7	6.4	19.7	35.4	14.3	7.7
improved	34.4	31.3	47.0	55.4	72.7	43.5
bush/field	53.9	62.3	33.2	9.1	13.0	48.8
Shared toilet with other households						
n	6500	5586	6323	8161	7044	4374
yes	63.8	50.7	66.6	42.6	31.9	70.5
no	36.2	48.7	33.4	57.4	68.1	29.5
don’t know	0.0	0.6	0.0	0.0	0.0	0.0
Hand washing place inside the house						
n	14,156	14,424	9671	9510	8380	9529
observed place	55.1	80.9	87.0	71.2	48.5	83.5
not observed place	44.9	19.1	13.0	28.8	51.5	16.5
Frequency any household member smoking						
n	14,156	14,424	9681	9510	8380	9544
never	85.3	72.2	74.2	77.5	70.4	83.7
daily	8.5	21.3	21.8	16.2	19.3	13.5
weekly	5.4	4.5	2.9	4.5	3.2	2.1
monthly	0.4	0.6	0.5	0.9	2.5	0.3
less than once a month	0.4	1.3	0.6	0.9	4.2	0.4
don’t know	0.0	0.0	0.0	0.0	0.4	0.0

n = Sample size used for tabulation; N = Total of sample size

^a^low pollution fuels include electricity, liquefied petroleum gas (LPG), natural gas and biogas

^b^medium pollution fuels include kerosene, coal/lignite and charcoal

^c^high pollution fuels include wood, straw/shrub/grass, agricultural crops and animal dung

### Children’s characteristics

Children under-five’s characteristics of interest are summarized in Table 2. This is a population with an average age of around 2 years, with a slight male predominance except for the Ivory Coast (51.2% girls versus 48.8% boys). Low birth weight, responsible for children's greater vulnerability to diseases, is observed with relatively low prevalence. Indeed, highest prevalence is noted in Burkina Faso (8.4%). As for symptoms of ARI, highest prevalence is noted in Togo, Ivory Coast and Burkina Faso for respectively 51.3%, 42.7% and 39.6%. As regards to their mothers, they were on average between 28 and 30 years old and are mostly uneducated except Togo (Benin: 65.6%; Burkina Faso: 83.9%; Ivory Coast: 63.9%; Mali: 73.2%; Senegal: 61.1%; Togo: 40.8%).

**Table 2:** WAEMU countries children under 5’s characteristics including symptoms of Acute Respiratory Illness (ARI), low birth weight and their mother’s highest education and tobacco use

Children and their mother’s characteristics	Benin	Burkina Faso	Ivory Coast	Mali	Senegal	Togo
	*N* = 12,432	*N* = 13,583	*N* = 6941	*N* = 9222	*N* = 11,182	*N* = 6405
Mother's age in years						
n	12,432	13,583	6941	9222	11,182	6405
mean	29.24	29.22	28.56	28.80	30.15	30.11
95% CI						
lower	29.07	29.05	28.30	28.57	29.95	29.85
upper	29.41	29.40	28.83	29.02	30.35	30.36
Mother's educational level						
n	12,432	13,583	6941	9222	11,179	6405
higher	1.3	0.5	1.0	1.3	2.1	1.6
secondary	14.9	4.7	9.3	13.8	13.7	21.1
primary	18.1	10.9	25.9	11.7	22.6	36.5
no education	65.6	83.9	63.9	73.2	61.6	40.8
don’t know	0.0	0.1	0.0	0.0	0.0	0.0
Does the mother smokes cigarettes						
n	12,432	13,583	6934	9222	11,182	6405
yes	1.6	0.1	0.2	0.8	0.7	0.0
no	98.4	99.9	99.8	99.2	99.3	100.0
Children’s current age in year						
n	12,432	13,583	6941	9222	11,182	6405
mean	1.92	1.95	1.901	1.93	1.99	1.95
95% CI						
lower	1.90	1.93	1.86	1.90	1.96	1.92
upper	1.94	1.96	1.94	1.95	2.01	1.98
Children’s Sex						
n	12,432	13,583	6941	9222	11,182	6405
male	50.6	50.5	48.8	50.7	51.2	50.1
female	49.4	49.5	51.2	49.3	48.8	49.9
Low birth weight infant^a^						
n	12,432	13,583	6880	9222	11,182	6392
yes	7.0	8.4	8.3	5.2	7.2	5.7
no	93.0	91.6	91.7	94.8	92.8	94.3
Whether the child had suffered from rapid breathing						
n	12,432	1407	1418	9222	11,182	1675
yes	7.6	40.2	44.3	6.1	8.8	52.0
no	91.5	58.5	54.6	93.0	88.3	48.0
don’t know	0.9	1.3	1.0	0.9	2.8	0.0
Whether the child has a problem in the chest or a blocked or running nose						
n	938	597	596	513	1019	903
chest only	18.0	12.5	10.6	11.4	16.0	7.5
nose only	59.0	51.5	59.0	64.9	46.5	74.6
both	19.6	34.6	27.0	21.2	33.6	16.7
other	0.8	0.5	1.9	0.2	3.6	0.2
don't know	2.6	1.0	1.6	2.3	0.3	1.0
Symptoms of Acute Respiratory Illness (ARI)^b^						
n	938	597	596	513	1019	903
yes	7.4	39.6	42.7	6.0	8.5	51.3
no	92.6	60.4	57.3	94.0	91.5	48.7

^a^Live births who were weighed at birth and were reported as weighing less than 2.5 kg

^b^Short, rapid breathing which was chest-related and/or difficult breathing which was chest-related

### Household levels of air pollution based on combustion process

**Fig. 1:**
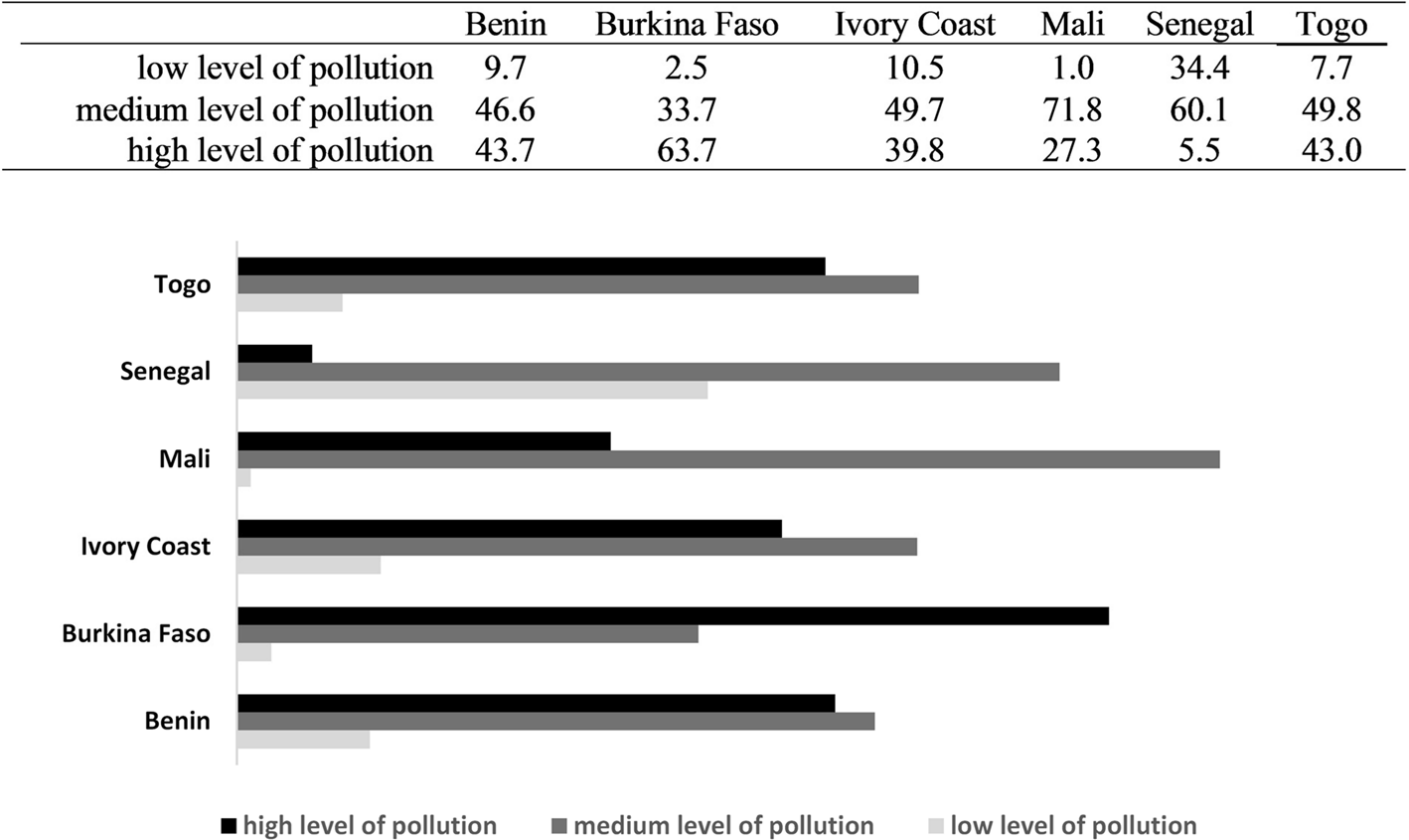
WAEMU countries household levels of air pollution based on combustion process

The main exposure factor “*Household level of air pollution*” is used to classify households into *low*, *medium* and *high* levels of indoor air pollution. This approach allowed to obtain frequencies summarized in Fig. 1. We can thus see a greater proportion of households with a high level of pollution in Burkina Faso (63.7%) followed by Benin (43.7%) then Togo (43.0%). %). As for Mali, Senegal and Ivory Coast, households are mainly characterized by a medium level of pollution: frequencies noted are respectively equal to 71.8%, 60.1% and 49.7%.

### Association measurement between symptoms ARI and children’s selected characteristics

Table 3 summarizes measures of association between symptoms of ARI and the main exposure variable “*Household level of air pollution*”. Other variables described in the literature as factors associated with ARIs are also included in analysis. The main exposure factor was only associated with occurrence of ARI in Togo (*p-value* < 0.001). Moreover, considering all countries included in the study, variables significantly associated with ARI are: Place of residence (Senegal: *p-value* = 0.046; Togo: *p-value* < 0.001), main source of drinking water (Togo: *p-value* = 0.012), type of toilet (Benin: *p-value* = 0.036; Senegal: *p-value* = 0.006), handwashing equipment (Senegal: p -value < 0.0001), mother’s age (Benin: *p-value* = 0.008) and also child’s age (Benin: *p-value* < 0.001; Ivory Coast: *p-value* = 0.034; Mali: *p* -value = 0.006; Senegal: *p-value* < 0.001; Togo: *p-value* = 0.026).

**Table 3:** Bivariate association between Symptoms of Acute Respiratory Illness (ARI) and selected characteristics of children and households in WAEMU countries

Variables	Benin		Burkina Faso		Ivory Coast		Mali		Senegal		Togo	
	**Symptoms of Acute Respiratory Illness (ARI) in % (column)**											
	yes	no	yes	no	yes	no	yes	no	yes	no	yes	no
Household level of air pollution												
low level of pollution	8.5	9.8	3.2	2.2	9.4	11.3	1.3	1.0	33.4	34.5	6.9	7.6
medium level of pollution	44.1	46.8	31.3	35.3	50.8	48.9	69.7	71.9	60.5	60.1	44.6	55.3
high level of pollution	47.4	43.4	65.5	62.5	39.8	39.8	29.0	27.1	6.1	5.4	48.5	37.1
* p*-value	0.194		0.356		0.718		0.636		0.720		** < 0.001**	
Type of place of residence												
urban	30.3	39.7	26.5	26.5	38.6	42.5	18.5	20.8	41.8	36.8	34.9	46.1
rural	69.7	60.3	73.5	73.5	61.4	57.5	81.5	79.2	58.2	63.2	65.1	53.9
* p*-value	** < 0.001**		0.999		0.420		0.586		**0.046**		** < 0.001**	
Source of drinking water												
unimproved	36.3	32.4	24.0	20.5	21.6	24.0	34.0	32.3	18.9	20.1	40.4	32.0
improved	63.7	67.6	76.0	79.5	78.4	76.0	66.0	67.7	81.1	79.9	59.6	68.0
* p*-value	0.105		0.253		0.570		0.692		0.482		0.012*	
Location of source for water												
in own dwelling/yard/plot	15.2	15.7	3.5	3.7	16.1	17.6	19.4	16.4	11.3	13.8	7.1	8.2
elsewhere	84.8	84.3	96.5	96.3	83.9	82.4	80.6	83.5	88.7	86.2	92.9	91.8
* p*-value	0.801		0.880		0.709		0.321		0.249		0.530	
Type of toilet facility												
unimproved	14.8	12.6	6.3	8.4	20.3	17.0	40.7	36.0	17.0	19.2	8.7	8.7
improved	23.2	28.7	34.0	34.9	39.8	46.2	49.1	54.4	71.7	65.6	37.1	43.8
bush/field	62.0	58.7	59.7	56.8	39.9	36.8	10.2	9.6	11.4	15.2	54.3	47.5
* p*-value	**0.036**		0.501		0.396		0.433		**0.006**		0.064	
Shared toilet with other households												
yes	70.5	64.8	45.2	52.3	58.5	65.0	35.2	43.4	26.5	23.8	70.3	73.3
no	29.5	35.2	54.8	47.7	41.5	35.0	64.8	56.6	73.5	76.2	29.7	26.7
* p*-value	0.062		0.126		0.194		0.075		0.203		0.484	
Hand washing place inside the house												
observed place	54.5	56.6	78.2	80.9	55.6	51.7	66.6	68.7	51.9	41.9	16.2	17.0
not observed place	45.5	43.4	21.8	19.1	44.4	48.3	33.4	31.3	48.1	58.1	83.8	83.0
* p*-value	0.393		0.437		0.416		0.617		** < 0.0001**		0.7157	
Mother's age in years												
< 18	1.5	0.8	1.4	0.8	2.6	2.5	1.9	2.0	1.2	1.0	0.8	1.2
18—24	27.6	30.1	30.0	30.1	29.3	31.0	24.2	27.1	25.3	21.4	20.6	20.3
25—34	52.7	44.5	46.8	44.5	47.1	46.5	46.7	48.8	50.1	50.2	55.0	52.5
> 34	18.3	24.6	21.8	24.6	21.0	20.0	27.2	22.0	23.4	27.4	23.6	25.9
* p*-value	**0.008**		0.594		0.958		0.204		0.065		0.726	
Mother's educational level												
no education	66.7	65.5	77.2	78.7	57.8	56.9	72.2	73.2	57.1	62.0	44.1	37.4
higher	1.0	1.4	0.6	1.3	1.3	1.5	1.8	1.3	2.3	2.1	1.9	1.8
secondary	13.5	15.0	9.9	8.2	12.4	11.3	15.5	13.7	16.2	13.4	18.2	24.0
primary	18.8	18.1	12.3	11.9	28.5	30.3	10.5	11.8	24.4	22.4	35.8	36.7
* p*-value	0.564		0.652		0.925		0.693		0.206		0.059	
Children’s current age												
< 1	32.7	22.3	24.2	23.5	25.6	18.8	23.9	21.5	27.1	19.3	23.0	18.3
1—2	24.1	19.5	27.8	26.9	22.6	27.2	25.4	21.2	23.8	20.5	26.5	24.6
2 – 3	17.4	18.7	21.3	22.4	20.9	21.9	19.7	18.2	21.3	19.5	18.0	23.0
≥ 3	25.8	39.6	26.8	27.3	30.9	32.1	31.0	39.1	27.9	40.7	32.4	34.1
* p*-value	** < 0.001**		0.957		**0.034**		**0.006**		** < 0.001**		**0.026**	
Children’s Sex												
male	51.0	50.5	53.9	52.9	47.9	47.4	48.9	50.8	50.6	51.3	49.5	49.9
female	49.0	49.5	46.1	47.1	52.1	52.6	51.1	49.2	49.4	48.7	50.5	50.1
* p*-value	0.815		0.756		0.889		0.517		0.756		0.859	
Low birth weight infant												
yes	5.9	7.0	11.3	9.8	9.3	8.9	4.7	5.2	8.0	7.1	6.1	6.8
no	94.1	93.0	88.7	90.2	90.7	91.1	95.3	94.8	92.0	92.0	93.9	93.2
* p*-value	0.296		0.403		0.843		0.638		0.465		0.584	

### Impact measurement by logistic regression

Multivariate models’ parameters are summarized in Table 4 below. The impact was mixed and not significant overall. Indeed, exposure to high level of pollution is associated with a non-significant risk only in Ivory Coast, Senegal and Togo. Adjusted Odds Ratios (AOR [95% CI]) are respectively estimated at 1.29 [0.72–2.30], 1.39 [0.94–2.05] and 1.15 [0.67–1.95] for these countries. Moreover, children under 2 years from rural households where handwashing place is not available are at a higher risk of developing ARI symptoms.

**Table 4:** Multivariate logistic regression between ARI and household level of air pollution, adjusted for some others covariables

**Variables**	**Benin**			**Burkina Faso**			**Ivory Coast**		
	AOR	95% CI	*P*	AOR	95% CI	*P*	AOR	95% CI	*P*
Household level of air pollution									
low level of pollution^ref^	-			-			-		
medium level of pollution	0.83	[0.49–1.43]	0.51	0.71	[0.31–1.65]	0.427	1.28	[0.75–2.16]	0.364
high level of pollution	0.87	[0.50–1.53]	0.65	0.83	[0.35–1.99]	0.676	1.29	[0.72–2.30]	0.397
Type of place of residence									
urban^ref^	-			-			-		
rural	1.46	[1.16–1.84]	**0.001**	0.90	[0.60–1.35]	0.606	1.12	[0.74–1.71]	0.589
Source of drinking water									
improved^ref^	-						-		
unimproved	1.80	[0.87–1.34]	0.49	1.28	[0.90–1.83]	0.170	0.77	[0.47–1.25]	0.287
Type of toilet facility									
improved^ref^	-			-			-		
unimproved	1.32	[0.96–1.81]	0.084	0.78	[0.43–1.40]	0.405	1.40	[0.88–2.21]	71.7
bush/field	1.07	[0.83–1.40]	0.593	1.05	[0.73–1.50]	0.806	1.26	[0.70–2.25]	0.44
Hand washing place inside the house									
observed place^ref^	-						-		
not observed place	1.65	[1.15–2.36]	**0.007**	1.20	[0.78–1.85]	0.399	0.82	[0.56–1.20]	0.314
Mother's age in years									
> 35^ref^				-			-		
< 18	1.39	[0.72–2.62]	0.341	0.53	[0.17–1.59]	0.255	0.85	[0.32–2.25]	0.736
[18–25)	1.19	[0.94–1.50]	0.144	0.88	[0.61–1.26]	0.488	0.87	[0.58–1.29]	0.475
[25–35)	1.19	[0.95–1.48]	0.133	0.82	[0.60–1.16]	0.259	0.94	[0.62–1.42]	0.769
Children’s current age									
[2–3)^ref^	-			-			-		
< 1	1.56	[1.26–1.94]	** < 0.001**	1.09	[0.76–1.58]	0.631	1.46	[1.00–2.12]	**0.044**
[1–2)	1.30	[1.02–1.65]	**0.033**	1.08	[0.78–1.51]	0.615	0.86	[0.59–1.26]	0.445
≥ 3	0.70	[0.56–0.88]	**0.002**	1.04	[0.74–1.47]	0.801	0.99	[0.70–1.40]	0.936
Children’s Sex									
male^ref^	-			-			-		
female	0.98	[0.84–1.15]	0.835	0.97	[0.75–1.24]	0.782	0.95	[0.72–1.26]	0.733
**Variables**	**Mali**			**Senegal**			**Togo**		
	AOR	95% CI	*P*	AOR	95% CI	*P*	AOR	95% CI	*P*
Household level of air pollution									
low level of pollution^ref^	-			-			-		
medium level of pollution	0.21	[0.11–0.86]	**0.025**	1.16	[0.93–1.45]	0.179	0.81	[0.51–1.30]	0.387
high level of pollution	0.35	[0.12–1.03]	0.057	1.39	[0.94–2.05]	0.096	1.15	[0.67–1.95]	0.613
Type of place of residence									
urban^ref^	-			-			-		
rural	1.08	[0.64–1.82]	0.760	0.82	[0.64–1.04]	0.105	1.59	[1.05–2.39]	**0.028**
Source of drinking water									
improved^ref^	-			-			-		
unimproved	1.01	[0.71–1.44]	0.94	1.00	[0.79–1.27]	0.975	1.17	[0.84–1.63]	0.357
Type of toilet facility									
improved^ref^	-			-			-		
unimproved	1.22	[0.86–1.72]	0.263	0.80	[0.63–1.03]	0.088	0.79	[0.50–1.25]	0.31
bush/field	1.09	[0.68–1.75]	0.719	0.69	[0.52–0.91]	**0.009**	0.80	[0.54–1.19]	0.26
Hand washing place inside the house									
observed place^ref^	-			-			-		
not observed place	1.13	[0.73–1.75]	0.589	1.20	[0.90–1.61]	0.216	0.87	[0.60–1.27]	0.475
Mother's age in years									
> 35^ref^	-			-			-		
< 18	0.64	[0.30–1.38]	0.252	1.16	[0.62–2.15]	0.645	0.67	[0.19–2.29]	0.517
[18–25)	0.69	[0.47–1.01]	0.057	1.25	[0.94–1.65]	0.123	1.10	[0.78–1.56]	0.595
[25–35)	0.76	[0.55–1.04]	0.081	1.11	[0.88–1.40]	0.372	1.25	[0.91–1.71]	0.175
Children’s current age									
[2–3)^ref^	-			-			-		
< 1	1.04	[0.78–1.39]	0.769	1.26	[0.98–1.61]	0.075	1.59	[1.12–2.24]	**0.009**
[1–2)	1.13	[0.86–1.49]	0.385	1.04	[0.84–1.28]	0.741	1.41	[1.02–1.94]	**0.037**
≥ 3	0.72	[0.54–0.96]	**0.028**	0.63	[0.51–0.79]	** < 0.001**	1.20	[0.90–1.61]	0.217
Children’s Sex									
male^ref^	-			-			-		
female	1.08	[0.85–1.37]	0.513	1.01	[0.86–1.19]	0.896	0.99	[0.80–1.22]	0.919

*ref* Reference category

## Discussion

This study assessed the level of indoor air pollution in WAEMU area and measured its impact on the respiratory health of children under five. The study’s approach is not based on air quality metrology, but rather on the use of data from Demographic and Health Surveys. In order to carry out this work, two main variables were defined: one as a dependent variable (symptoms of ARI) and the other is considered as the main exposure factor “*Household level of air pollution*”. Among the eight WAEMU countries, two were excluded from the study: Guinea Bissau(not part of the DHS program) and Niger for which some variables on combustion processes were not available in the database.

Results reveal a predominance of rural households with a good standard of living with regard to the combined wealth index and mainly, using highly polluting fuels for cooking activities (Benin: 65.9%; Burkina Faso: 87.7%; Ivory Coast: 60.2%; Mali: 77.9%; Togo: 49.4%). Access to clean cooking is relatively low in these countries, proportions are below estimated averages in 2010 and 2019 for Sub-Saharan Africa (≈ 9% and 13%) and for Sahel (≈ 10% and 9%): 5.0% (Benin), 5.3% (Burkina Faso), 15.0% (Ivory Coast), 1.1% (Mali), 28.4% (Senegal), 6.9% (Togo) [6]. The use of biomass (wood and charcoal) as cooking energy is indeed very widespread in WAEMU area. It is respectively estimated at: 61.9% and 26.7% (Benin); 87.5% and 4.3% (Burkina Faso); 60.1% and 17.6% (Ivory Coast); 77.6% and 19.3% (Mali); 0.0% and 18.9% (Senegal); 1.4% and 48.2% (Togo). The use of wood and charcoal are respectively estimated at: 61.9% and 26.7% (Benin); 87.5% and 4.3% (Burkina Faso); 60.1% and 17.6% (Ivory Coast); 77.6% and 19.3% (Mali); 0.0% and 18.9% (Senegal); 1.4% and 48.2% (Togo). These fuels are highly polluting due to the composition of the smoke they emit [29]. A large part of households in these countries use stoves where biomass is incompletely burnt due to oxygen deficit as a means of cooking [4, 7, 29]. Smoke from these stoves constitutes a heterogeneous mixture of chemical compounds in gaseous or particulate form known for their adverse effects on respiratory system [2, 29, 30]. Among main gaseous pollutants are nitrogen oxides (Nox), carbon monoxide (CO) and other volatile organic compounds (VOCs) [1, 29]. As for particulate pollutants, they are more than 90% inhalable (aerodynamic diameter < 10 mm) and consist in a complex mixture of organic and inorganic substances suspended in the air [1, 31]. Other works in West Africa have also dealt with the use of biomass in cooking activities and concluded with the following estimates: 72% (Benin); 60% (Burkina Faso); 73% (Ivory Coast); 98% (Mali); 52.3% (Senegal); 71% (Togo) [4, 15, 32–35]. These results seem to confirm Senegal as a country where biomass is used the least for cooking compared to other countries. In Senegal, households mainly use lignite (43.1%) and liquefied petroleum gas (27%) while in the other countries, the use of these same fuels is respectively estimated at: 1.0% and 3.3% (Benin); 0.1% and 4.6% (Burkina Faso); 0.2% and 14.8% (Ivory Coast); 0.4% and 0.3% (Mali); 42.1% and 6.5% (Togo). Moreover, results are in the same direction as those in the literature on Mali, a country which uses biomass the most as the main cooking fuel. In addition, cooking takes place mainly outdoor (Benin: 50.6%; Burkina Faso: 67.9%; Ivory Coast: 50.6%; Togo: 66.6%) with the exception of Mali, where it mainly takes place inside the home in a separate room (68.8%) and in Senegal, where it takes place mostly in the housing (71.3%). The place of cooking is indeed a determinant factor of indoor air quality. Due to poor ventilation, households without a separate kitchen from accommodation are exposed to higher levels of pollution [36].

Indoor air pollution is not from cooking activities alone, factors such as lighting and secondhand smoke can contribute to the degradation of indoor air quality [8, 37]. Indeed, in the absence of electricity, households mainly resort to polluting fuels such as kerosene, candles, etc. to ensure their lighting needs [8]. Results revealed a level of access to electricity in Senegal (63.3%) and in Ivory Coast (55.9%) higher than the average in Sub-Saharan Africa (50% in 2019) and in Sahel (43% in 2016) while more than half of households in other countries do not have access (Benin: 64.4%; Burkina Faso: 86.8%; Mali: 51.4%; Togo: 54.2%) [38]. As regards to tobacco, cigarettes in particular, when used in an oxygen-depleted space, its smoke can be comparable to that of biomass, it is composed of gas and suspended particles. Among other compounds found in this smoke, can be mentioned gases irritating respiratory tract such as nitrogen dioxide, sulfur dioxide and acrolein [37, 39]. Daily exposure to passive smoking is more accentuated in Burkina Faso (21.3%) and Ivory Coast (21.8%) compared to other countries (Benin: 8.5%; Mali: 16.2%; Senegal: 19.3%; Togo: 13.5%). These results are partially superimposable on the prevalence of tobacco use in WAEMU region: 14.5% (Burkina Faso), 14.1% (Mali), 10.1% (Ivory Coast), 9.0% (Benin), 6.6% (Senegal), 6.4% (Togo) [40], Burkina Faso and Togo have respectively the highest and lowest prevalence. Furthermore, cigarette smoking by mothers whose children were included in this study remains low and varies between 0.0% (Togo) and 1.6% (Benin). Indeed, in the WAEMU area, the overall prevalence of tobacco use by women (2.8%) is comparable to that in Sub-Saharan Africa (3%) and is relatively low compared to Europe and Central Asia (31%) as well as in North America (18%) [41].

Burkina Faso stands out with a greater number of households with a high level of pollution (63.7%) followed by Benin (43.7%) then Togo (43.0%). In Mali, Senegal and Ivory Coast, households are mainly characterized by a medium level of pollution (71.8%, 60.1% and 49.7% respectively). Few studies published on air quality in Africa have mainly focused on outdoor particulate matter pollution (PM_2.5_ and PM_10_) [14, 42]. These are generally used as an indicator of air quality [14]. PM_2.5_ are reported by some studies on indoor air quality in West Africa, and the documented levels were: 26.55.103 mg / m^3^ (Burkina Faso), 121 ± 12 μg / m^3^ and 32 ± 3 μg / m^3^ (Ivory Coast), 10.3 to 17.3 μg / m^3^ (Togo) [4, 15, 43]. As for PM_10_, they were recorded with levels varying between 11.6 to 18.4 μg / m^3^ (Togo) and 30 mg / m^3^ (Senegal) [43, 44]. Although these observations cannot be superimposed on the results, they seem to confirm Burkina Faso as the most affected country by indoor air pollution with regard to PM_2.5_’s level. Indoor air pollution is also a documented risk factor of ARI occurrence in children under five, which symptoms are more noted in Togo (51.3%) [11, 45]. The main exposure variable “*Household level of air pollution*” was associated with ARI symptoms (Togo: *chi-squared* test’s *p-value* < 0.001). Although the impact was not significant overall, exposure to a *high level* of pollution is associated with an slight risk in Ivory Coast, Senegal and Togo: the Adjusted Odds Ratios (AOR [95% CI]) on child’s age and sex, mother’s age, place of residence, main source of drinking water, type of toilet facility and availability of handwashing place, are respectively estimated at 1.29 [0.72–2.30], 1.39 [0.94–2.05] and 1.15 [0.67–1.95].. This could be the result of two potential explanations. At first, the infectious etiology of ARI, suggesting other factors such as indoor air pollution as an exacerbating factor [46], which condition is mainly spread under influence of poor hygiene and sanitation practices, by inhalation or by contact with body fluids/droplets charged with infectious agents (bacteria, viruses) [47]. Good Hygiene and Sanitation Practices (BPHA) cover handwashing with soap, effective use of latrines as well as preservation of the salubrity of water from point of draw to consumption [46, 48]. However, the results show that the risk of ARI is increase by 1.46 [1.16–1.84] (Benin) and 1.59 [1.05–2.39] (Togo) for children from rural households. Place of residence is indeed a major determinant of access to Water, Hygiene and Sanitation (WASH) [49] and the study reveals a predominance of rural households, many of which use unimproved drinking water sources and toilet facility. In Togo for example, 56% of households are settled in rural place, of which 32% use unimproved sources of drinking water and for most of these households, 89.5% of which source is not located in the dwelling. In addition, 43.5% of households use an improved toilet and in 70.5% of cases, the toilet is shared with other households. Second explanation concerns lower precision of indicator used to characterize indoor pollution compared to an indoor air metrology. In addition, operational definition of ARI has evolved over time and symptoms are self-reported. The present study defines symptoms of ARI as the presence in the child of “*short, rapid breathing which was chest-related and/or difficult breathing which was chest-related*” during two weeks preceding the survey. This is different from the following definition, used by most studies that have explored relationship between ARI and indoor air quality: “*cough accompanied by short and rapid breathing due to a lung problem and/or difficult breathing related to a lung problem*” [27, 28, 50].

Our study has a number of limitations. It is a cross-sectional study that did not allow a conclusion to be drawn from causal relationship between exposure factor and dependent variable, because its design did not take into account temporality of events. It is a secondary analysis of data from DHS surveys in West Africa, survey participants themselves reported information of interest. Therefore, it is difficult to eliminate some biases, especially those relating to the self-reporting. Data used were not collected during the same period, making it difficult to compare countries included in study. However, DHS remains one of the world's most well-developed primary sources of demographic and health data.

## Conclusion

This study reveals a predominance of rural households, cooking mainly in outdoor and using highly polluting fuels. Moreover, more than half of households in Togo, Benin, Burkina Faso and Mali do not have access to electricity. Although the majority of households are not exposed to second-hand smoke, prevalence of daily exposure varies between 8.5% and 21.8%. Households with a high level of pollution are more noted in Burkina Faso followed by Benin then Togo while in Mali, Senegal and Ivory Coast, households are mainly characterized by a medium level of pollution. As regards to ARI Symptoms, the prevalence is higher in Togo, Ivory Coast and Burkina Faso while in overall, the impact of “*Household level of air pollution*” is not significant. Indeed, only the place of residence, the age of the child and the availability of handwashing place at home remain significantly associated with the occurrence of ARI. These findings could serve as public health policy support and guide for further research on the impact of indoor air pollution on respiratory health in Africa.

## Data Availability

The data used for this article are accessible to researchers on condition of briefly explaining the purpose for it use. Details of the access conditions are available at the DHS program-http://dhsprogram.com address.

## Abbreviations

ARI: Acute Respiratory Illness
BPHA: Good Hygiene and Sanitation Practices
DHS: Demographic and Health Surveys
STIs: Sexually Transmitted Infections
LMICs: Low and Middle Income Countries
WAEMU: West African Economic and Monetary Union
WASH: Water, Hygiene and Sanitation
